# Histopathology and expression of the chemokines CXCL10, CXCL13, and CXCR3 and the endogenous TLR-4 ligand S100A8/A9 in lymph nodes of patients with adult-onset Still’s disease

**DOI:** 10.1038/s41598-019-44032-6

**Published:** 2019-05-17

**Authors:** Hyoun-Ah Kim, Yon Hee Kim, Yoon Kyung Jeon, Woo-Ick Yang, Ji Eun Kwon, Jae Ho Han

**Affiliations:** 1 Department of Rheumatology, Ajou University School of Medicine, Suwon, Korea; 2 Department of Pathology, Soonchunhyang University Hospital, Seoul, Korea; 3 Department of Pathology, Seoul National University College of Medicine, Seoul, Korea; 4 Department of Pathology, Yonsei University College of Medicine, Seoul, Korea; 5 Department of Pathology, Ajou University School of Medicine, Suwon, Korea

**Keywords:** Diagnostic markers, Connective tissue diseases

## Abstract

Adult-onset Still’s disease (AOSD) is a rare systemic inflammatory disease manifesting with a persistent high-spiking fever, a typical rash, and lymphadenopathy. Endogenous factors related to interleukin-1, such as S100A8/A9 and several chemokines including CXCL10, CXCR3, and CXCL13, potentially play roles in its pathogenesis. We describe the histopathological features and chemokine expression pattern in lymph nodes (LNs) of patients with AOSD. Formalin-fixed, paraffin-embedded excisional LN tissues from 48 patients with AOSD were histologically reviewed. CXCL10, CXCR3, CXCL13, and S100A8/A9 expression was evaluated immunohistochemically. The pathology of LN was characterized by paracortical hyperplasia with proliferation of histiocyte, immunoblast, CD8-positive lymphoid cell and blood vessel. Most cases required differential diagnosis from dermatopathic lymphadenitis (n = 16, 33.3%), T cell lymphoma (n = 11, 22.9%), and histiocytic necrotizing lymphadenitis (HNL) (n = 9, 18.8%). The expression levels of CXCL10 and CXCR3 were higher in patients with AOSD than in those with T cell lymphoma, HNL, tuberculous lymphadenitis, and reactive hyperplasia. It is important to recognize the aforementioned histopathologic findings of nodal involvement of AOSD because improper diagnosis and treatment can be avoided. Immunohistochemical staining for chemokines, CXCL10 and CXCR3, may aid in differentiating AOSD from other mimickers.

## Introduction

Adult-onset Still’s disease (AOSD), first described in 1971 by Bywaters, is a rare systemic inflammatory disorder of unknown etiology^1^. The typical features include a high-spiking fever, a salmon-like rash, and arthritis. Although patients complain of various clinical manifestations, including sore throat, lymphadenopathy, abdominal pain, and pericarditis, no pathognomic or diagnostic test is available^1,2^. The laboratory features are variable; only a high serum ferritin level is specific. Prognosis varies by disease course and organs involved. Although various etiological factors, including infection, a genetic propensity, and immunological disorders, have been suggested, the pathogenesis of AOSD remains poorly understood^3^. Innate immune reactions mediated by macrophages, monocytes, and T cells are in play; these cells secrete well-known proinflammatory cytokines and chemokines, including interleukin (IL)-1, IL-6, IL-8, IL-18, interferon-γ (IFN-γ), and tumor necrosis factor-α^3,4^. Previously, we used immunohistochemistry (IHC) to show that the expression levels of CXCL10, CXCR3, and CXCL13 were elevated in skin biopsy specimens from 26 patients with AOSD. The serum levels of CXCL10 and CXCL13 serve as markers of disease activity^5^. S100A8/A9, an endogenous ligand of Toll-like receptor-4, has also been used as a disease marker and plays an important role in AOSD pathogenesis^4^.

Lymphadenopathy is not rare in patients with AOSD, being present in 0–90% of these individuals^2,6,7^. However, previous studies of the histological features of lymph nodes (LNs) enrolled only small numbers of patients, most of whom exhibited the paracortical pattern of reactive hyperplasia in the LNs^7–11^. The paracortical pattern is characterized by paracortical proliferation of immunoblasts and increased density of high endothelial venules. Histological examination of LNs is performed principally to rule out hematological malignancy or infectious diseases, which has symptoms similar to those of AOSD and is also diagnostically ambiguous^7^. In some earlier cases, the original pathological diagnoses were lymphomas, especially angioimmunoblastic T-cell lymphoma (AITL)^6–8^. We previously described the typical pathological findings for many organs in 32 patients with AOSD and for the LNs in 8 of these patients. Unlike other organs, the LNs were invaded by more CD4-positive than CD8-positive lymphocytes^9^. Another study of the LNs of 12 patients with AOSD showed that the CD4:CD8 cell ratio was approximately 3:2^10^. However, the studies enrolled small numbers of patients. To our knowledge, no study has yet measured chemokine levels in the LNs of patients with AOSD. Here, we describe the typical histological findings of LNs; we used IHC to detect CXCL10, CXCR3, CXCL13, and S100A8/A9 and compared their levels of LN of AOSD patients with those of other diseases.

## Results

### Clinical characteristics of the patients

Table 1 summarizes the clinical characteristics of the 48 patients with AOSD. The mean age was 41.5 ± 14.3 years and 95.8% of patients were female. The clinical symptoms included high-spiking fever (97.9%), skin rash (93.8%), arthralgia (75.0%), sore throat (52.1%), and splenomegaly (47.9%). The mean levels of ferritin, erythrocyte sedimentation rate (ESR) and C-reactive protein (CRP) were 11,142.7 ± 13,727.0 ng/mL, 61.5 ± 30.4 mm/hr and 15.8 ± 24.1 mg/dL. The laboratory test and LN biopsies of all AOSD patients were done in the initial stages of high-level disease activity before treatment. The mean systemic score was 6.3. Five (10.4%) patients died from recurrence and complications 10 days to 11 years after enrollment.

**Table 1:** Clinical characteristics of patients.

	AOSD (n = 48)
Age (years)	41.5 ± 14.3
Gender (F/M)	46 (95.8)/2 (4.2)
Fever	47 (97.9)
Sore throat	25 (52.1)
Skin rash	45 (93.8)
Lymphadenopathy	48 (100)
Splenomegaly	23 (47.9)
Hepatomegaly	17 (35.4)
Pericarditis	4 (8.3)
Pleuritis	11 (22.9)
Pneumonia	9 (18.8)
Arthralgia	36 (75.0)
Hemoglobin, g/dL	10.3 ± 1.8
Leukocytes, /μL	13,841.7 ± 8,530.6
Platelets, ×10^3^/μL	282.5 ± 146.7
Ferritin, ng/mL	11,142.7 ± 13,727.0
ESR, mm/h	61.5 ± 30.4
CRP, mg/dL	15.8 ± 24.1
AST/ALT, mg/dL	193.3 ± 303.6/174.6 ± 496.1
Bilirubin, mg/dL	0.6 ± 0.65
LDH, U/L	412.35 ± 298.9
Systemic score	6.3 ± 1.4
Alive/dead	43 (89.6)/5 (10.4)

AOSD, adult-onset Still’s disease; ESR, erythrocyte sedimentation rate; CRP, C-reactive protein; AST, aspartate transaminase; ALT, alanine transaminase; ANA, antinuclear antibody; RF, rheumatoid factor; LDH, lactate dehydrogenase. All values are presented as numbers (with percentages) or as means ± standard deviations. The systemic scoring system of Pouchot *et al*.^24^ assigns a score from 0 to 12 with 1 point for each of the following manifestations: fever, the typical rash, pleuritis, pneumonia, pericarditis, hepatomegaly or abnormal liver function test data, splenomegaly, lymphadenopathy, leukocyte count ≥ 15,000/mm^2^, sore throat, myalgia, and abdominal pain.

### Histopathological characteristics of LNs

**Figure 1:**
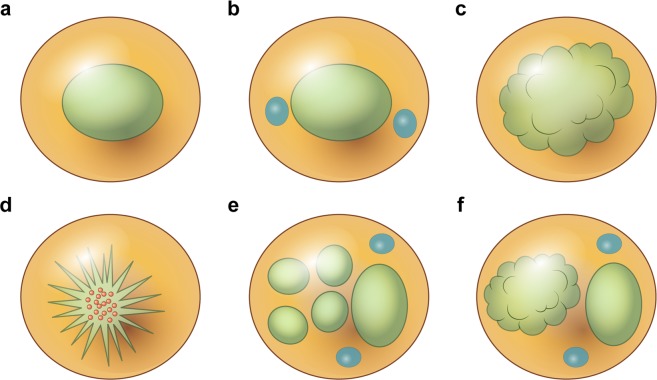
Schematic diagrams of the histological reaction patterns of lymph nodes: (**a**) follicular, (**b**) paracortical, (**c**) diffuse, (**d**) necrotizing, (**e**,**f**) mixed, (**e**) is correspond to mixed follicular and paracortical and (**f**) mixed diffuse and paracortical. Green color indicates predominant lesional area: blue color, residual normal lymphoid follicle: red color, karyorrhexis.

**Figure 2:**
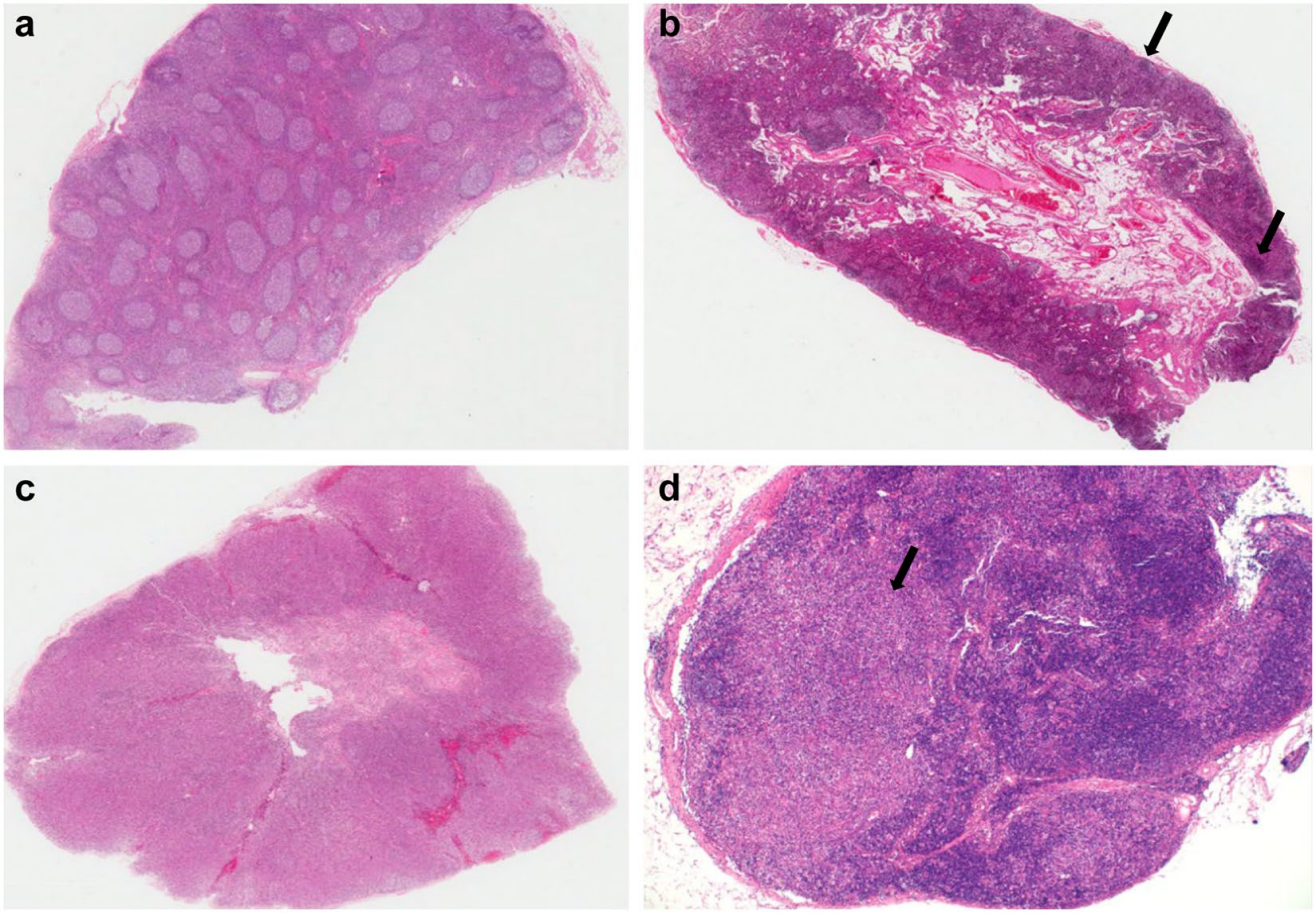
Hematoxylin and eosin staining of the several distinctive patterns of lymph nodes of patients with adult-onset Still’s disease. (**a)** Follicular pattern (×12.5), (**b**) paracortical pattern (×12.5), (**c**) diffuse pattern (×12.5), (**d**) necrotizing pattern (×12.5). Note numerous lymphoid follicle with germinal center throughout the lymph node in follicular pattern (**a**) and tiny residual lymphoid follicle (arrow) and expanded paracortex in paracortical pattern (**b**). In diffuse pattern (**c**), the lymphoid follicle is not found. In necrotizing pattern, discrete area of necrosis (arrow) and adjacent expanded paracortex are noted (**d**). Original magnification, x12.5 (**a**–**d**)

**Figure 3:**
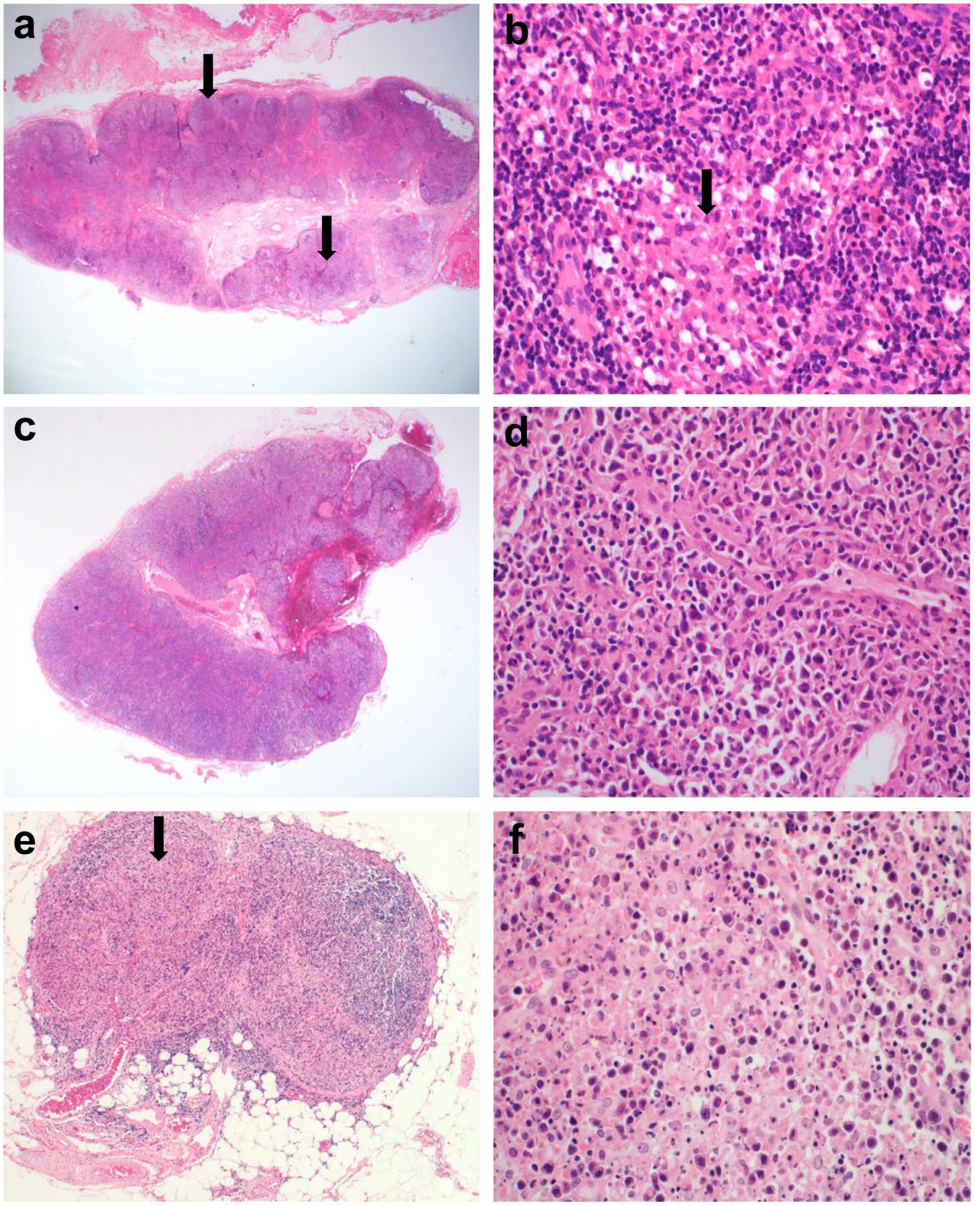
Three histological types requiring differential diagnosis: (**a**,**b**) dermatopathic lymphadenitis, (**c,d**) lymphoma, and (**e**,**f**) histiocytic necrotizing lymphadenitis. Note nodular expansion of paracortex (arrow, **a**) with aggregation of histiocytes (arrow, **b**). Diffuse effacement of lymph node architecture (**c**) by infiltration of atypical cells (**d**). Pale area of necrosis (arrow, **e**) with a mixture of karyorrhectic nuclear debri, single cell necrosis and a few large lymphoid cells (**f**). Original magnification, x12.5 (**a**,**c**,**e**), x400 (**b**,**d**,**f**).

Common patterns were paracortical (n = 17, 39.6%) and mixed (n = 17, 35.4%), followed by diffuse (n = 8, 19.0%), and necrotizing (n = 4, 8.3%), and follicular (n = 2, 4.2%, Table 2, Figs 1 and 2). The infiltrating inflammatory cells were mainly eosinophils and histiocytes. Eosinophil infiltration was evident in 54.2% of samples. Histiocytic proliferation was moderate to severe in almost all (95.8%) cases. About half of the patients evidenced karyorrhexis and six showed necrosis. Some of these cases (18.8% of the total sample) were categorized as mimics of histiocytic necrotizing lymphadenitis (HNL) (Fig. 3e,f). Twenty-three (48.0%) patients exhibited moderate to severe immunoblastic proliferation and 32 (66.7%) patients showed moderate to severe vascular proliferation.

**Table 2:** Histological features of lymph nodes (LNs) in 48 patients with adult-onset Still’s disease (AOSD).

		AOSD, n (%)
Pattern of reaction	Follicular	2 (4.2)
	Paracortical	17 (35.4)
	Diffuse	8 (19.0)
	Mixed	17 (35.4)
	Necrotizing	4 (8.3)
Differential diagnosis	Dermatopathic lymphadenitis	16 (33.3)
	Lymphoma	11 (22.9)
	Non-specific hyperplasia	9 (18.8)
	Infectious mononucleosis	3 (6.3)
	Histiocytic necrotizing lymphadenitis	9 (18.8)
Immunoblast proliferation	Mild, <5/HPF	25 (52.1)
	Moderate, 5–10/HPF	9 (18.8)
	Severe, >10/HPF	14 (29.2)
Histiocytic proliferation	Mild, <5/HPF	2 (4.2)
	Moderate, 5–10/HPF	23 (47.9)
	Severe, >10/HPF	23 (47.9)
Plasmacytic infiltration	Absent	42 (87.5)
	Present	6 (12.5)
Eosinophilic infiltration	Absent	22 (45.8)
	Present	26 (54.2)
Neutrophilic infiltration	Absent	45 (93.8)
	Present	3 (6.3)
Hemophagocytosis	Absent	45 (93.8)
	Present	3 (6.3)
Necrosis	Absent	42 (87.5)
	Present	6 (12.5)
Karyorrhexis	Absent	28 (58.3)
	Present	20 (41.7)
Vascular proliferation	Mild	16 (33.3)
	Moderate	20 (41.7)
	Severe	12 (25.0)

HPF: high-power field.

The histological features of dermatopathic lymphadenitis (DL) include nodular expansion of the pale-staining paracortical zones with proliferation of macrophages containing melanin pigment. Cases with the diffuse pattern featuring immunoblast proliferation, proliferation of high endothelial venules, and infiltration of atypical cells were mimics of lymphoma. Sixteen (33.3%) cases exhibited DL patterns (Fig. 3a,b), and 11 (22.9%) cases showed lymphoma patterns (Fig. 3c,d).

### Comparison of CXCL10, CXCR3, CXCL13 and S100A8/A9 expression in LNs of 41 patients with AOSD and those with T cell lymphoma, HNL, tuberculous (TB) lymphadenitis, and nonspecific reactive hyperplasia

**Figure 4:**
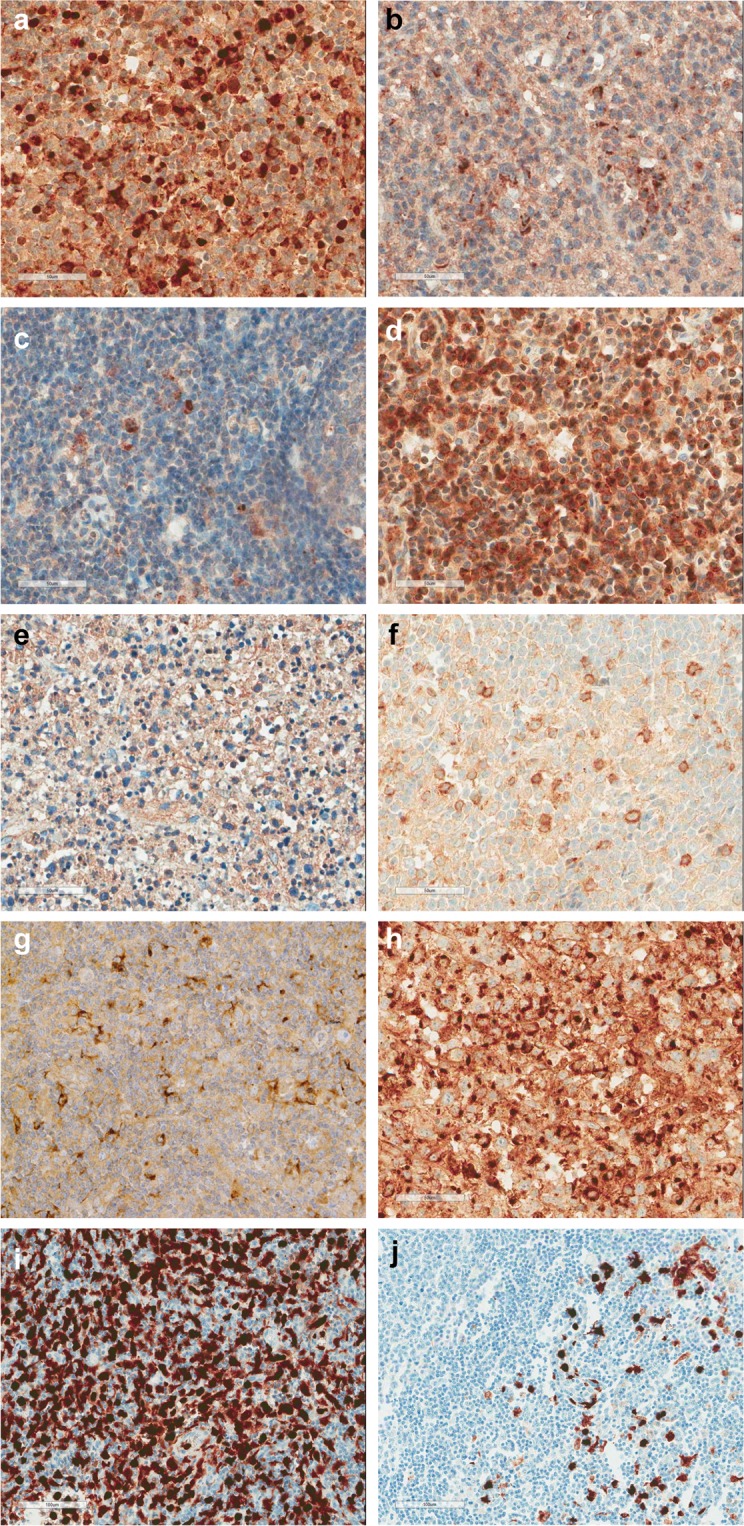
Immunohistochemical staining for CXCL10 (**a**–**c**), CXCR3 (**d**–**f**), CXCL13 (**g**,**h**) and S100A8/A9 (**i**,**j**) from adult-onset Still’s disease (AOSD) (**a**,**d**,**g**,**i**,**j**), T cell lymphoma (**b**,**h**), histiocytic necrotizing lymphadenitis (**e**) and reactive hyperplasia (**c**,**f)**. Representative examples of frequent (**a,d,h**,**i**) and rare expression (**b**,**c**,**e**–**g**,**j**). CXCL10 and CXCR3 are frequently expressed while CXCL13 are rarely expressed in lymph node from patients with AOSD. Original magnification, x400 (**a**–**h**), x200 (**i**,**j**).

**Figure 5:**
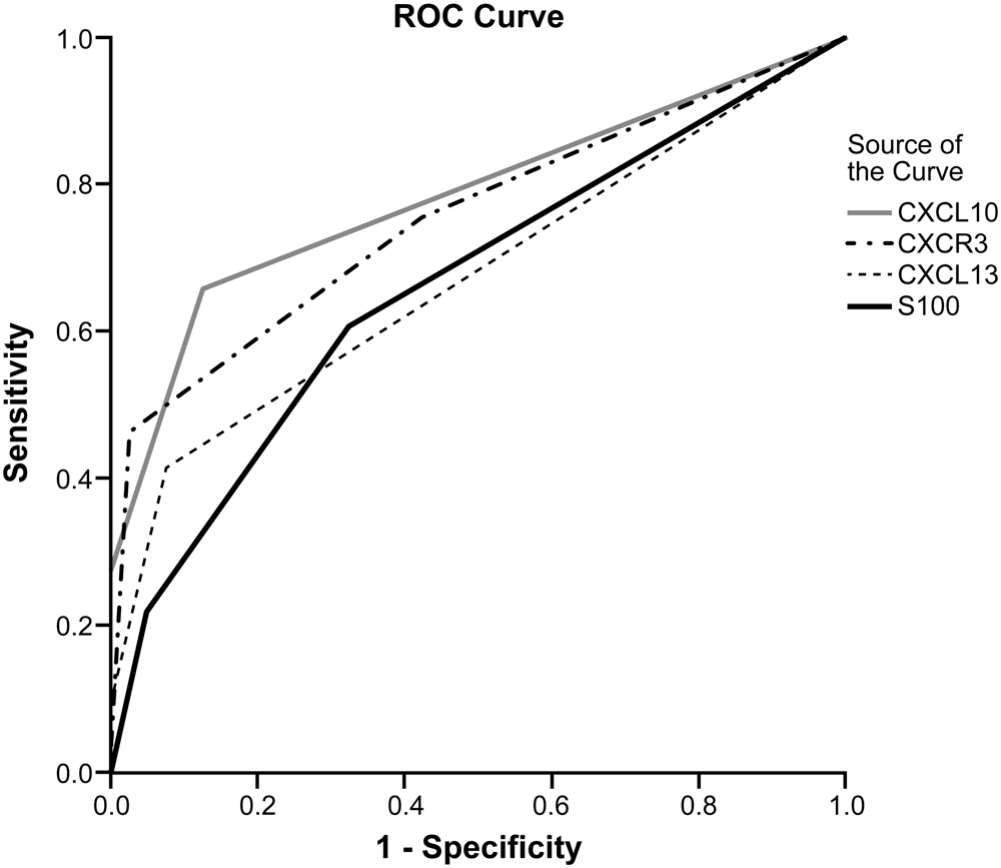
The receiver-operating characteristic (ROC) curves for CXCL10, CXCR3, CXCL13 and S100A8/A9 expressing inflammatory cells in lymph node of adult-onset Still’s disease (AOSD) and controls (T cell lymphoma, histiocytic necrotizing lymphadenitis, tuberculous lymphadenitis, and reactive hyperplasia). The ROC curve values were 0.784 (95% CI 0.681–0.886, p < 0.001) for CXCL10, 0.755 (95% CI 0.648–0.861, p < 0.001) for CXCR3, 0.602 (95% CI 0.477–0.726, p = 0.116) for CXCL13, and 0.663 (95% CI 0.544–0.781, p = 0.061) for S100A8/A9.

The IHC findings for CXCL10, CXCL13, CXCR3, and S100A8/A9 are shown in Table 3 and Fig. 4. CXCL10-expressing inflammatory cells were of grade 1 in 14 (34.1%) AOSD cases, grade 2 in 16 (39.0%), and grade 3 in 11 (26.8%) cases. CXCL10-expressing lymphoid cells were of grade 1 in 9 (90.0%) T cell lymphoma cases, and grade 2 in only 1 (10.0%) case. CXCL10-expressing inflammatory cells were of grade 1 in 6 (60.0%), and grade 2 in 4 (40.0%) cases of HNL and TB lymphadenitis. All cases of reactive hyperplasia were of grade 1 in terms of CXCL10-expressing inflammatory cells. The CXCL10 grade was significantly higher in patients with AOSD than in those with T cell lymphoma, HNL, TB lymphadenitis, and reactive hyperplasia (p < 0.001). CXCR3-expressing inflammatory cells were of grade 1 in 10 (24.4%) AOSD cases, grade 2 in 12 (29.3%), and grade 3 in 19 (46.3%) cases. Six (60.0%) cases of T cell lymphoma were of grade 1, three (30.0%) were of grade 2, and only one (10.0%) was of grade 3. Seven (70.0%) cases of HNL were of grade 1, and three (30.0%) were of grade 2. Six (60.0%) cases of reactive hyperplasia were of grade 1 and four (40.0%) were of grade 2. The CXCR3 grade was also higher in patients with AOSD than in those with T cell lymphoma, HNL, TB lymphadenitis, and reactive hyperplasia (p = 0.002). Furthermore, the CXCL13 grades were different significantly among the five groups (p = 0.004). However, the number of S100A8/A9-positive inflammatory cells was somewhat increased in the LNs of patients with AOSD, but statistical significance was not attained (p = 0.273). In a Receiver-Operating Characteristics (ROC) analysis of the chemokines and S100A8/A9-expressing inflammatory cells, area under the curve (AUC) was 0.784 (95% CI 0.681–0.886, p < 0.001) for CXCL10, 0.755 (95% CI 0.648–0.861, p < 0.001) for CXCR3, 0.602 (95% CI 0.477–0.726, p = 0.116) for CXCL13, and 0.663 (95% CI 0.544–0.781, p = 0.061) for S100A8/A9 (Fig. 5).

**Table 3:** Comparison of immunohistochemical staining data from 41 patients with adult-onset Still’s disease (AOSD) and those with T cell lymphoma (TCL) (n = 10), histiocytic necrotizing lymphadenitis (HNL) (n = 10), and tuberculous (TB) lymphadenitis (n = 10), and reactive hyperplasia (n = 10).

	AOSD	TCL	HNL	TB lymphadenitis	Reactive hyperplasia	*P*-value
**CXCL10**						<**0**.**001**
Grade 1	14 (34.1)	9 (90.0)	6 (60.0)	6 (60.0)	10 (100)	
Grade 2	16 (39.0)	1 (10.0)	4 (40.0)	4 (40.0)	0 (00.0)	
Grade 3	11 (26.8)	0 (00.0)	0 (00.0)	0 (00.0)	0 (00.0)	
**CXCR 3**						**0**.**002**
Grade 1	10 (24.4)	6 (60.0)	7 (70.0)	4 (40.0)	6 (60.0)	
Grade 2	12 (29.3)	3 (30.0)	3 (30.0)	6 (60.0)	4 (40.0)	
Grade 3	19 (46.3)	1 (10.0)	0 (00.0)	0 (00.0)	0 (00.0)	
**CXCL13**						**0**.**004**
Grade 1	24 (58.8)	3 (30.0)	10 (100.0)	10.0 (100.0)	9 (90.0)	
Grade 2	13 (31.7)	4 (40.0)	0 (00.0)	0 (00.0)	1 (10.0)	
Grade 3	4 (9.8)	3 (30.0)	0 (00.0)	0 (00.0)	0 (00.0)	
**S100A8/A9**						0.273
Grade 1	16 (39.0)	7 (70.0)	7 (70.0)	7 (70.0)	6 (60.0)	
Grade 2	17 (41.5)	2 (20.0)	2 (20.0)	3 (30.0)	4 (40.0)	
Grade 3	8 (19.5)	1 (10.0)	1 (10.0)	0 (00.0)	0 (00.0)	

These data are shown to n (%). The LN chemokine expression levels in patients with AOSD and those with TCL, HNL, TB lymphadenitis, and reactive hyperplasia were compared using the chi-squared test for categorical variables.

Samples from the 11 AOSD patients exhibiting diffuse and mixed patterns that were mimics of lymphoma were subjected to additional IHC staining to rule out lymphomas. The typical morphological features of AITL include follicular dendritic cell proliferation around the high endothelial venules or outside the germinal centers; we immunostained for PD-1 and CD21. None of the 11 cases stained for CXCL13 or PD-1, unlike AITL. CXCL13 and PD-1 was expressed by only small scattered lymphoid cells in lymphoid follicles and the paracortical areas of LNs from patients with AOSD. Follicular dendritic cell meshworks expressing CD21 were not expanded in patients with AOSD with the mixed pattern and were absent in those with the diffuse pattern.

### A correlation of serum concentrations (S100A8/A9 and the other cytokines) and expression in lymph nodes

Serum chemokines and S100A8/A9 levels with LN biopsies were obtained from 8 patients with AOSD. Serum mean CXCL10 level was 920.3 ± 1463.9 pg/mL, mean CXCL13 level 158.4 ± 100.7 pg/mL, mean S100A8/A9 15.6 ± 7.9 μg/mL. However, serum CXCL10 levels were not correlated with the percentage of inflammatory cells expressing CXCL10 (r = -0.368, p = 0.345). Serum CXCL13 levels were not correlated with the percentage of inflammatory cells expressing CXCL13 (r = 0.169, p = 0.689). Also, serum S100A8/A9 levels were not correlated with the percentage of inflammatory cells expressing S100A8/A9 (r = 0.110, p = 0.795).

### Associations among histopathological characteristics, clinical manifestations, and chemokine levels in LNs of patients with AOSD

The chemokine-positive grades were compared with the histopathological characteristics of AOSD LNs. The CXCL10 grade was higher in LNs exhibiting karyorrhexis than in those that did not (p = 0.022, data not shown), and in LNs exhibiting necrosis than in those that did not (p = 0.029). The CXCL13 grade was higher in LNs exhibiting karyorrhexis and necrosis than in those that did not (p < 0.001, p = 0.006). Furthermore, CXCL13 expression was associated with immunoblastic proliferation (p = 0.034). However, the CXCR3 grade did not differ by karyorrhexis or necrosis status. The S100A8/A9 grade was higher in LNs exhibiting karyorrhexis and necrosis than in those that did not (p = 0.013 and p = 0.018, respectively).

In terms of clinical manifestations, patients with arthralgia had higher CXCR3 grades than did those without arthralgia (p = 0.020). Furthermore, patients with pericarditis had higher CXCL13 grades than did those without pericarditis (p = 0.043). The CXCL10 and S100A8/A9 grades of LNs were not associated with any clinical AOSD manifestation (hepatosplenomegaly, pericarditis, or skin rash).

## Discussion

The diagnosis of AOSD with LN involvement is challenging for clinicians and pathologists. The pathogenesis, disease activity, and histological features of AOSD remain under active research. We found that the LNs of patients with AOSD exhibited paracortical expansion with infiltration of polymorphic inflammatory cells, including small to medium-sized lymphocytes, immunoblasts, epithelioid histiocytes, eosinophils, and small numbers of plasma cells and neutrophils, similar to previous studies^8,11–13^. Furthermore, we found that the CXCL10 and CXCR3 levels were higher in the LNs of patients with AOSD than in those of patients with T cell lymphoma, HNL, TB lymphadenitis and reactive hyperplasia. CXCR3 and CXCL13 may serve as predictive markers of clinical manifestations, including pericarditis and arthralgia, in patients with AOSD. Although these chemokines are presently of limited diagnostic utility, this may change in the future.

Common disorders that are difficult to differentiate from AOSD lymphadenopathy include HNL and AITL; these three conditions are histopathologically similar. We review the literature on differential diagnosis and our experiences with IHC.

First, HNL is a self-limiting disease featuring subacute, necrotizing regional lymphadenopathy associated with mild fever, sore throat, and myalgia in young Asians. The characteristic histological features of HNL include aggregations of histiocytes, plasmacytoid monocytes, and activated T lymphocytes associated with patchy necrosis, nuclear debris, and marked apoptosis^14^. The infiltration of neutrophil is not found. The histopathology of LNs in HNL is similar, and has been suggested to differentiate the conditions from AOSD^9^. In the present study, almost all LNs of patients with AOSD exhibited moderate to severe histiocytic proliferation. Therefore, histiocytic proliferation cannot differentiate the two diseases. In addition, only three (6.3%) of our patients with AOSD exhibited neutrophilic infiltration, and 20 (41.7%) had non-neutrophilic karyorrhexis.

Previous studies revealed abundant T cells (more CD8+ than CD4+ cells) and non-neutrophilic karyorrhexis in the LNs of patients with HNL^15–17^. In our study, 25 (61%) AOSD patients had more CD8+ than CD4+ cells. One study evaluated the expression levels of IFN-γ, CXCL10, and CXCR3 in lymphadenopathic tissues from patients with HNL; CXCR3-positive lymphocytes were frequently detected in the surrounding dead areas, as were CXCL10-positive cells^18^. In this study comparing chemokine expression between patients with AOSD and those with HNL, the CXCR3 expressing level in LNs was higher in patients with AOSD than in those with HNL. Previous studies have shown that AOSD and HNL are related, suggesting that co-occurrence may shed light on the pathological mechanisms shared by the two diseases^19,20^. Therefore, IHC analysis for these chemokines could be additional biomarkers for differentiation between the two diseases. Further additional pathophysiological research is needed.

Second, lymphadenopathy in patients with AOSD may be confused with malignant lymphoma clinically and even histologically. AITL is an especially difficult differential diagnosis; the clinical manifestations and histological features are similar. AITL is a lymphoma of mature follicular helper T cells and typically presents with systemic symptoms, generalized lymphadenopathy and hepatosplenomegaly. Other common findings are skin rash, arthritis, pleural effusion and ascites^21^. Although the primary site of disease is the LNs, the spleen (50–70%), liver (50–70%), skin (50%), pleuropulmonary system (40%), and bone marrow (15–40%) may be involved. The clinical course is aggressive; the median survival time is <3 years. The morphological features of AITL include partial effacement of the nodal architecture, marked proliferation of arborizing endothelial venules, and a predominantly paracortical polymorphic infiltrate composed of small to medium-sized lymphocytes exhibiting minimal cytologic atypia. Therefore, the differential diagnosis of AOSD and AITL by microscopy is difficult. The neoplastic cells of AITL can express some antigens of follicular T-helper cells, such as CD10, BCL-6, CXCL13, ICOS, SLAM-associated protein, and PD-1. These antigens are helpful indistinguishing AITL from atypical paracortical hyperplasia in AOSD. An inverse correlations tend to be observed between sensitivity and specificity of individual markers, with CXCL13 and CD10 being the most specific and PD1 and ICOS being more sensitive. To identify diagnostic tools distinguishing AOSD and AITL, we performed IHC for CXCL10, CXCL13, CXCR3, S100A8/A9, PD-1 and CD21, which are particularly useful markers for the diagnosis of AITL^22^. In previous studies, the lymphoma cells from all the lymph node with AITL were positive for both PD-1 and CXCL13^22^, but PD-1 was expressed more prominently (in terms of percentage and intensity) than CXCL13. Neoplastic cells also expressed CD3/CD4, but not CD8. One study of only three LNs of patients with AOSD revealed that two (66.7%) cases were more positive for CD4 than CD8^11^. Although the CD4/CD8-positive lymphocyte ratio is relatively low (about 3:1) in patients with AOSD, those with AITL exhibited predominantly CD4-positive interfollicular T cells that were polymorphic in pattern. In our previous study, the number of CD4-positive lymphocytes was greater than that of CD8-positive lymphocytes in seven of the eight (87.5%) cases; the ratio was about 2–3:1^9^. However, in the present study, the number of CD8-positive lymphocytes was greater than that of CD4-positive cells in more than half (61.0%) of LNs from patients with AOSD. Moreover, 7 of 11 (63.3%) patients with the lymphoma pattern expressed more CD8 than CD4. Unlike the LNs of patients with AITL, for which the addition of an anti-CD21 antibody highlights the extensive, follicular dendritic cell proliferation, CD21 expression in all LNs from patients with AOSD was confined to normal follicular dendritic cells of the germinal centers. Furthermore, CXCL10-expressing inflammatory cells of patients with AOSD were higher than those of patients with T cell lymphoma. Thus, IHC for CD21, PD1, CD4/CD8, and CXCL10 usefully differentiates the two diseases, accelerating diagnosis and avoiding unnecessary treatment.

A previous study found no association between the clinical features and LN patterns of patients with AOSD^10^. Only two patients with pronounced S100-positive histiocytosis in the LNs exhibited elevated serum ferritin values. Here, we also found no association between LN pathological patterns and AOSD clinical manifestations. However, CXCR3 expression in LNs was associated with arthralgia and pericarditis in patients with AOSD, suggesting that the chemokine was associated with these conditions.

Our study has certain limitations. First, our work was retrospective in nature; some patient data were missing. Second, serum samples from only 8 AOSD patients could be obtained for evaluation of correlation between serum chemokine levels and LN chemokine expression. Further prospective studies with larger samples are required to fully assess the utility of LN histology and the levels of various chemokines in patients with AOSD.

In conclusion, the histopathological findings of AOSD are sufficiently diverse to require several differential diagnoses; the typical histological features are paracortical hyperplasia with prominent histiocytes and immunoblasts; CD8+ -predominant, small to medium lymphocytic infiltration; and vascular proliferation. The CXCL10 and CXCR3 expression levels are higher than in patients with several lymphadenopathic diseases. The LN biopsy findings could be challenged to pathologists due to similarities between AOSD lymphadenopathy and HNL or AITL. It is important to recognize the aforementioned histopathologic findings and immunohistochemical staining results of nodal involvement of AOSD. After excluding the possibility of lymphoma or other autoimmune diseases, the pathologist could suggest the possibility of AOSD to the physician. Further studies are needed to identify biomarkers differentiating AOSD from several other diseases.

## Methods

### Subjects

We retrospectively included 48 patients with AOSD examined at the Seoul National University College of Medicine, the Yonsei University College of Medicine (Seoul), and the Ajou University School of Medicine between January 2004 and December 2012. All fulfilled the AOSD criteria of Yamaguchi *et al*.^23^. Only patients who underwent excisional LN biopsies were included. The exclusion criteria were specimen inadequacy (fibrous or fatty tissue content only, or poor quality rendering evaluation impossible) and repeat biopsy. We excluded lymphoma cases through re-examination of LN biopsy. The control groups were composed of 40 excisional LN samples from patients with lymphoma, HNL, TB lymphadenitis and nonspecific reactive hyperplasia. We reviewed clinical manifestations and laboratory findings. The clinical symptoms recorded were fever, the typical rash, pleuritis, pneumonia, pericarditis, hepatomegaly, splenomegaly, lymphadenopathy, sore throat, myalgia, and abdominal pain; the laboratory findings recorded were the complete blood count, erythrocyte sedimentation rate (ESR), levels of C-reactive protein (CRP) and ferritin, and liver function. AOSD disease activity was scored from 0 to 12, with 1 point allocated for each of fever, the typical rash, pleuritis, pneumonia, pericarditis, hepatomegaly or abnormal liver function, splenomegaly, lymphadenopathy, leukocyte count ≥15,000/mm^2^, sore throat, myalgia, and abdominal pain^24^. The Institutional Review Board of Ajou University Hospital approved the study (approval no. AJIRB-MED-15-472), which waived the requirement for informed consent because the LN data were analyzed retrospectively. Informed consent was obtained from 8 subjects for serum samples. All procedures for this study were carried out in accordance with the approved guidelines.

### Histopathological analysis of LNs

We examined hematoxylin and eosin–stained sections of excisional LN biopsy samples obtained from 48 patients with AOSD. Three pathologists (YHK, JEK, and JHH) independently examined all slides to determine: (1) the pattern (follicular, paracortical, diffuse hyperplasia, mixed, or necrotizing; Figs 1 and 2); (2) the differential diagnosis from HNL, lymphomas including AITL, DL, infectious mononucleosis, and nonspecific hyperplasia; (3) the types of infiltrating inflammatory cell; (4) the karyorrhexis and necrosis status; (5) the extents of immunoblastic and histiocytic proliferation (1: <5, 2: 5–10, 3: >10/high-power fields); and (6) the extent of vascular proliferation.

### Immunohistochemical staining for chemokines CXCL10, CXCL13, CXCR3, and S100A8/A9 in 41 patients with AOSD and 10 patients with T cell lymphoma, HNL, TB lymphadenitis, or reactive hyperplasia

Formalin-fixed, paraffin-embedded sections were analyzed by IHC using a Benchmark XT automated staining system (Ventana Medical Systems Inc., Tucson, AZ, USA). The primary antibodies used were [anti- (dilution factor; manufacturer)]: CD4 (prediluted; Roche, Basel, Switzerland), CD8 (prediluted; Roche), CD21 (prediluted; Roche), CXCL10 (1:30; R & D Systems, Minneapolis, MN, USA), CXCL13 (1:50; R & D Systems), CXCR3 (1:20; R & D Systems), S100A8/A9 (1:900; Abcam, Cambridge, UK), and programmed death-1 (PD-1, 1:50, Cell Marque, Rocklin, CA, USA). Staining was detected using a Ventana Optiview DAB kit (Ventana Medical Systems Inc.). The results for CXCL10, CXCL13, CXCR3 and S100A8/A9 IHC were graded from 1 to 3 according to the percentage of positive lymphoid cells and histiocytes (1: 1–33%, 2: 34–66%, 3: 67–100%).

### Serum levels of chemokines CXCL10, CXCL13, and S100A8/A9

Serum from 8 patients with AOSD were obtained. Their levels were evaluated with commercial enzyme-linked immunosorbent assay (ELISA) kits according to the manufacturer’s instructions (CXCL10 and CXCL13 from R & D systems, Minneapolis, MN, USA, and S100A8/A9 from Buhlmann Laboratories, Schonenbuch, Switzerland).

### Statistical analyses

Continuous variables are expressed as means ± standard deviations, and categorical variables are expressed as frequencies with percentages. The chemokine expression levels in LNs from patients with AOSD and those with AITL, HNL, TB lymphadenitis, and reactive hyperplasia were compared using the chi-squared test for categorical variables. Also, the ability of each chemokine and S100A8/A9 expression to accurately diagnose lymphadenopathy of AOSD was evaluated by a ROC analysis. The expression levels in LNs were compared according to AOSD clinical manifestations (including arthritis and skin rash) using the chi-squared test. We calculated Spearman correlations between the histological scores of LNs and systemic disease activity levels. All statistical analyses were performed using SPSS version 20.0 (SPSS, Chicago, IL, USA). P values < 0.05 were considered to indicate statistical significance.

## Data Availability

The datasets analyzed during the current study are available from the corresponding author on reasonable request.
